# Isolation and Characterization of *Lactobacillus crispatus*, *Lactococcus lactis*, and *Carnobacterium divergens* as Potential Probiotic Bacteria from Fermented Black and Green Olives (*Olea europaea*): An Exploratory Study

**DOI:** 10.1155/2023/8726320

**Published:** 2023-04-26

**Authors:** Ayesha Saeed, Azra Yasmin, Mehreen Baig, Khalid Khan, Md Belal Bin Heyat, Faijan Akhtar, Zahra Batool, Abeer Kazmi, Abdul Wahab, Muhammad Shahid, Muhammad Arslan Ahmed, Sidra Abbas, Abdullah Y. Muaad, Amir Shahzad, Imtiaz Ahmad

**Affiliations:** ^1^Microbiology and Biotechnology Research Lab, Fatima Jinnah Women University Rawalpindi, Pakistan; ^2^Surgical Unit II, Foundation University, Islamabad, Pakistan; ^3^Foot and Mouth Disease Vaccine Research Centre, Veterinary Research Institute (VRI), Peshawar, Pakistan; ^4^IOT Research Centre, College of Computer Science and Software Engineering, Shenzhen University, Shenzhen, Guangdong 518060, China; ^5^Centre for VLSI and Embedded System Technologies, International Institute of Information Technology, Hyderabad, Telangana 500032, India; ^6^Department of Science and Engineering, Novel Global Community Educational Foundation, Hebersham, NSW 2770, Australia; ^7^School of Computer Science and Engineering, University of Electronic Science and Technology of China, Chengdu, China; ^8^Institute of Innovative Development of Food Industry, Shenzhen University, Shenzhen 518060, China; ^9^Shenzhen Key Laboratory of Marine Microbiome Engineering, Institute for Advanced Study, Shenzhen University, Shenzhen 518060, China; ^10^Institute of Hydrobiology, Chinese Academy of Sciences, University of Chinese Academy of Sciences (UCAS), Wuhan, China; ^11^University of Chinese Academy of Sciences, Beijing 100049, China; ^12^Shanghai Center for Plant Stress Biology, CAS Center for Excellence in Molecular Plant Sciences, Chinese Academy of Sciences, Shanghai 200032, China; ^13^Brucellosis Section, Veterinary Research Institute (VRI), Peshawar, Pakistan; ^14^Critical Care Medicine, Shifa International Hospital, Islamabad, Pakistan; ^15^Sana'a Community College, Yemen; ^16^Nishtar Medical University, Multan, Punjab, Pakistan; ^17^Medical Officer, Regional Health Centre (RHC), Qadirabad, Tehsil Kot Chutta, District Dera Ghazi Khan, Punjab, Pakistan

## Abstract

**Background:**

Table olives are becoming well recognized as a source of probiotic bacteria that might be used to create a health-promoting fermented food product by traditional procedures based on the activities of indigenous microbial consortia present in local environments. *Methodology*. In the present study, the characterization of probiotic bacteria isolated from mince, chunks, and brine of fermented green and black olives (*Olea europaea*) was done based on morphological, biochemical, and physiological characteristics.

**Results:**

Bacterial isolates demonstrated excellent survival abilities at 25, 37, and 45°C and at a variable range of pH. However, the optimum temperature is 37 and the optimum pH is 7 for all three isolates. An antimicrobial susceptibility pattern was found among these isolates through the disc diffusion method. Most of the isolates were susceptible to streptomycin, imipenem, and chloramphenicol, whereas, amoxicillin showed resistance to these isolates, and variable results were recorded for the rest of the antibiotics tested. The growth of the isolates was optimum with the supplementation of 3% NaCl and 0.3% bile salt. The isolated bacteria were able to ferment skimmed milk into yogurt, hence making it capable of producing organic acid.

**Conclusion:**

Isolates of *Lactobacillus crispatus* MB417, *Lactococcus lactis* MB418 from black olives, and *Carnobacterium divergens* MB421 from green olives were characterized as potential candidates for use as starter cultures to induce fermentation of other probiotic food products.

## 1. Introduction

Probiotics have a long history of human consumption; for example, cultured dairy products (curds and yogurts) are traditionally consumed in several parts of the world. The word probiotics is of Greek origin, meaning “for life”, which is the antonym of antibiotics [1, 2]. Those living microorganisms that are administered in an adequate amount and have a beneficial effect on human health are known as probiotics [3, 4].

According to Ranadheera et al., [5] and Oleskin, & Shenderov [6], the human gastrointestinal tract contains more bacteria than eukaryotic cells. This gut flora also contains probiotics, which constructively influence the body by offering health encouragement to the host [4].

Microorganisms used as probiotics, specifically lactic acid bacteria (LAB), include *Lactobacillus* species, some species of *Bifidobacteria, Enterococcus, and Streptococcus* [7, 8]. Most of these bacterial species reside in the human intestine [9]. The only probiotic yeast that exists is the nonpathogenic *Saccharomyces boulardii* [10–12]. LAB has prevalent exploitation in fermented food manufacturing [13] and is a generally recognized as safe (GRAS) organism that can be securely used for medical or veterinary purposes [14, 15].

If LAB is exposed to traumatic circumstances in the gastrointestinal tract like acidic gastric juice, bile salt, and/or altered microbial balance of the intestinal tract, then administration of any kind of antibiotic may suppress the probiotic population. So, to get the maximum benefits out of the probiotic bacterial population, the selection of probiotics is made after various *in vitro* and *in vivo* tests [16, 17].

There are a variety of traditional fermented food products produced by probiotic fermentation like fruits and vegetables including olives, beetroot, cabbage, and other leafy vegetables. Fermentation is done by keeping the vegetables in a 2% brine solution and allowing the stored vegetables to be fermented with LAB [18, 19]. In the food industry, especially for ready-to-eat foods, microbiological quality is a persistent concern. Lactic acid bacteria (LAB) could be used to prevent the growth of spoiling and pathogenic bacteria [20]. Some LAB exhibits active microbial antagonism through competition mechanisms, the production of bacteriocins, or the production of organic acids [21–23].

The plant named “olive” is a small tree of the family *Oleaceae*, and its binomial name is *Olea europaea* [24, 25]. Olives are bitter when raw or fresh, so they must be treated and fermented to make them edible. Both green olives, which are full-size olives plucked before ripening, and black olives, which are completely matured, ripened olives, can be fermented. The main reasons for olive processing are the exclusion of bitterness by hydrolysis of some phenolic compounds (like oleuropein), preservation of the fruit, and enhancement of the organoleptic characteristics of the ultimate product [26, 27].

Due to the high concentration of dietary fiber, vitamins, antioxidants, and anticancer chemicals in table olives, they have therefore been considered useful food [28]. Table olives are called pickled vegetables, in which preparation and maintenance are achieved by an amalgamation of salting, fermentation, and acidification. Some studies have been conducted to widen the range of useful food types by utilizing of the microarchitecture of the surface of olives, and the dietary characteristics of olive pulp to produce a flavorsome, vegetable-based efficient food comprising of table olives equipped with probiotic strains [29].

In research, the capacity of seven strains from the probiotic species *Lactobacillus rhamnosus*, *Lactobacillus paracasei*, *Bifidobacterium umbifidum*, and *Bifidobacterium longum* to survive on the olive surface and the suitability of table olives as a biological carrier for probiotic microorganisms were studied [30]. The resultant table olives can be stored with or without refrigeration, and probiotic-dominated fermentations are generally considered the most suitable method of curing olives [31, 32]. This study is aimed at identifying and functionalizing the characterization of isolated probiotics from commercially available green and black olives.

## 2. Materials and Methods

The research work was done in the Microbiology & Biotechnology Laboratory at Fatima Jinnah Women University, Rawalpindi. The work is divided into two phases: isolation and identification of bacteria and physiological characterization of probiotic isolates.

### 2.1. Sample Collection

Fermented pitted Spanish green and black olive samples of the same brand (Figaro Company) were purchased from local superstores located in the Pakistan Aeronautical Complex (PAC), Karma, and Rawalpindi. The sealed fermented green and black olives bottles were opened in sterile condition near a flame to avoid contamination. Using sterile forceps, a few olives from the bottles were taken out and then minced inside a mortar and pestle after disinfecting it with an ethanol (70%) swab. Before being minced, big chunks of both black and green olives were kept inside sterile universal bottles with the help of sterile forceps; later, the olive mince from the mortar was also collected in universal bottles and infused in autoclaved distilled water using a sterile spatula. The brine from black and green olive samples was also stored in a sterile universal bottle following the method of Doulgeraki et al. [33] with slight workable modifications. All the universal bottles containing olive and brine samples were stored in the refrigerator for isolation.

### 2.2. Isolation of Probiotics

Strains from olives were isolated on selective de Man, Rogosa, and Sharpe (MRS) media (Merck Millipore Germany, catalog # 110660) [34, 35]. Isolations from chunks, brine, and minced fermented black and green olives were done on MRS media by the spread plate method [36]. Isolates were characterized by morphological, biochemical, and physiological characteristics.

### 2.3. Morphological and Biochemical Characterizations

Morphological characterization was studied through the gram staining technique by Hans Christian Gram [37]. The catalase test [38], Simmons citrate test [39, 40], methyl red and Voges–Proskauer's test [41], indole production test [40, 42], and oxidation fermentation test [43, 44] were performed for biochemical characterization of the isolates. The identification of the isolated bacteria was done by the Standard API 50-CHL system [45].

### 2.4. Physiological Characterization

#### 2.4.1. Determination of Optimal Temperature

Isolated bacteria were incubated at 25°C, 37°C, and 45°C for 24 hours to determine their optimal temperature for growth, and the results were calculated by the spectrometric method at 600 nm [46].

#### 2.4.2. Determination of Optimal pH

Fresh bacterial cells were grown for 24 hours at pH ranges of 4–9 to determine whether the isolates were acidophilic, neutrophilic, or alkaliphilic. Optical density was measured by spectroscopic readings at 600 nm [46].

#### 2.4.3. The Antibiotic Sensitivity Assay

An antibiotic susceptibility test was conducted using the disc diffusion method [47]. Fresh overnight cultures of bacterial isolates were spread onto Mueller-Hinton (MH) agar media plates (recommended by the National Committee for Clinical Laboratory Standards (NCCLS, CLSI (2018)), and 10 antibiotic discs were placed on the media plates and incubated at 37°C for 24 h. The antibiotics used were amoxicillin (10 *μ*g), gentamicin (10 *μ*g), streptomycin (10 *μ*g), tetracycline (30 *μ*g), kanamycin (30 *μ*g), imipenem (10 *μ*g), chloramphenicol (30 *μ*g), bacitracin (10 *μ*g), erythromycin (15 *μ*g) and neomycin (30 *μ*g).

#### 2.4.4. Antimicrobial Activity

The antimicrobial activity of all isolates against indicator bacteria was determined by the agar-well diffusion method [48]. *Pseudomonas geniculate*, *Microbacterium oxydans*, *Bacillus subtilis*, *Streptomyces laurentii*, *Klebiella pneumonia*, *Bacillus pumilus*, *Bacillus cereus*, *Acaligens facelis*, *Enterococcus faecium*, and *Enterococcus facelis* were used as indicator bacteria (obtained from the Microbiology & Biotechnology Lab, FJWU) against which the antimicrobial activity of isolated strains was assayed. Each test string was inoculated into 5 ml of nutrient broth and incubated at 37°C for 24 hours on a shaking incubator at 150 rpm. After incubation, each culture was centrifuged at 10000 rpm for 5 minutes to obtain a cell-free supernatant. Supernatants of isolated species were identified for antibacterial activity besides indicator bacteria. For antimicrobial activity identification, Mueller-Hinton agar medium (for antimicrobial testing) was prepared, autoclaved, and poured separately into the sterile Petri dishes. By the spread plate method, the plates were inoculated with indicator bacterial suspension. Five wells, each with an 8 mm diameter, were generated in every nutrient agar plate, and the base of each well was sealed with soft agar (0.7%) plugs. To identify the antibacterial activity of probiotic isolates, 100 *μ*l of cell-free supernatants of probiotic strain was added to the well. The plates were incubated for 24 h at 37°C, and the diameter of the zone of inhibition was measured in millimeters on both nutrient agar and MH agar media.

#### 2.4.5. Salt Tolerance Assay

Isolate tolerance to NaCl was determined by supplementing nutrient broth with varying salt concentrations ranging from 1 to 11% [49].

#### 2.4.6. Organic Acid Production Assay

Lactic acid bacteria are known for the production of organic acids, specifically lactic acids. Isolates were considered to have the same property, and to determine this ability, an acid production assay was conducted [14]. The brine in which fermented green olives and black olives were stored was also assayed for organic acid produced by the isolated strains inhabiting the brine at the beginning, in the middle, and at the end of the research work. Powdered skim milk was purchased from the local market. Autoclaved distilled water was taken and mixed with 10% of powdered skim milk to make sterile skim milk with a pH of 6.68. Five ml of skimmed milk was inoculated with a 24 h fresh culture of isolated bacteria and incubated at 37°C for 24, 48, and 72 h. After incubation, coagulated skim milk was filtered, and the pH of each filtrate was measured with a digital electrode pH meter for lactic acid production. The filtrate was also titrated with 0.1 N NaOH, and organic acid production was quantified in terms of percentage strength.

#### 2.4.7. Bile Salt Tolerance Assay

The ability of isolated species to survive and grow in the presence of bile salts was investigated by growing the isolated strains with different bile salt concentrations (0.1, 0.3, 0.5, and 1%) in nutrient broth as described by Dunne et al. *[*47*]*. The broth was incubated for 4 h at 37°C, and the optical density (OD) of the cultures was then measured at 600 nm. The viability and growth of isolates at this concentration showed tolerance of isolated strains within the gastrointestinal tract of humans [51, 52].

### 2.5. Tolerance to Simulated Gastric Juice

Stimulated gastric juice tolerance was determined by the method described by Graciela and Maria [52]. Stimulated gastric juice was freshly prepared and sterilized by filter sterilization.

### 2.6. Numerical Taxonomy of Probiotic Isolates

Similarity among isolates was checked by taking data from fifty (50) different biochemical sugar fermentation tests from the API CH 50 kit and converting them into binary data (0 or 1) for negative or positive test results, respectively, using PAST (Paleontological Statistics Software Package for Education and Data Analysis software). Similarities amongst the strains were estimated using the Jacquard coefficient, and the unweighted average linkage gave the cluster.

## 3. Results

### 3.1. Isolation and Identification of Potential Probiotic Bacteria

**Figure 1:**
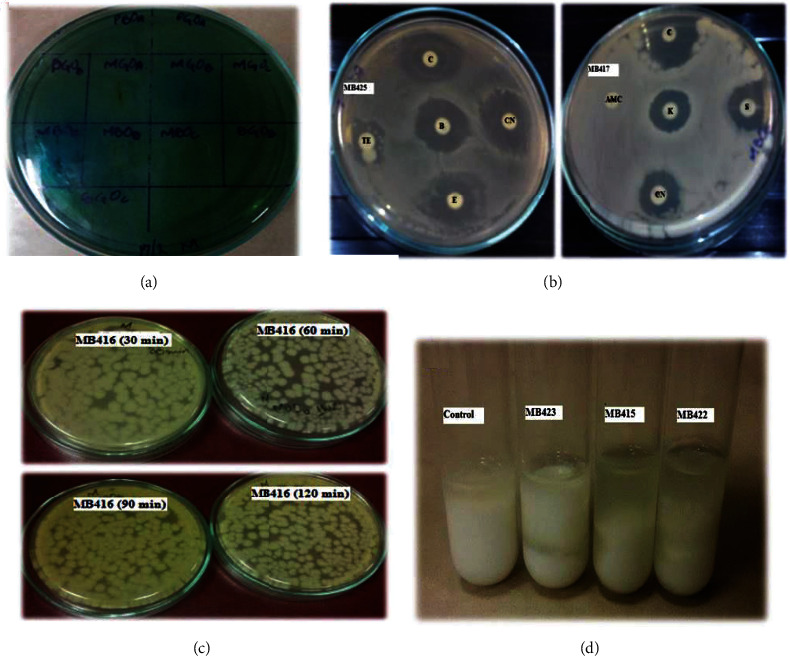
Different tests performed on isolated probiotic bacteria. (a) Culture of isolated probiotic bacteria on Simmon citrate agar. (b) Disc diffusion assay of MB425 and MB417. (c) Gastric juice tolerance test for MB416. (d) Milk coagulation by different isolates.

In the present study, probiotics were isolated from commercially available fermented black and green olives from the Figaro Company. Fermenter probiotic bacteria were isolated on an MRS medium, and a total of 12 isolates were obtained from brine, chunks, and suspension from minced olives (Figure 1). As shown in Table 1, potential probiotic bacteria were isolated from black and green olives. Identification was done by cell and colony morphology, which showed diversity ranging from 0.5 to 11.5 mm in size, circular to irregular shape, and white to pale color. All the isolated probiotic bacteria were stained as Gram-positive, and most of them were rod-shaped.

**Figure 2:**
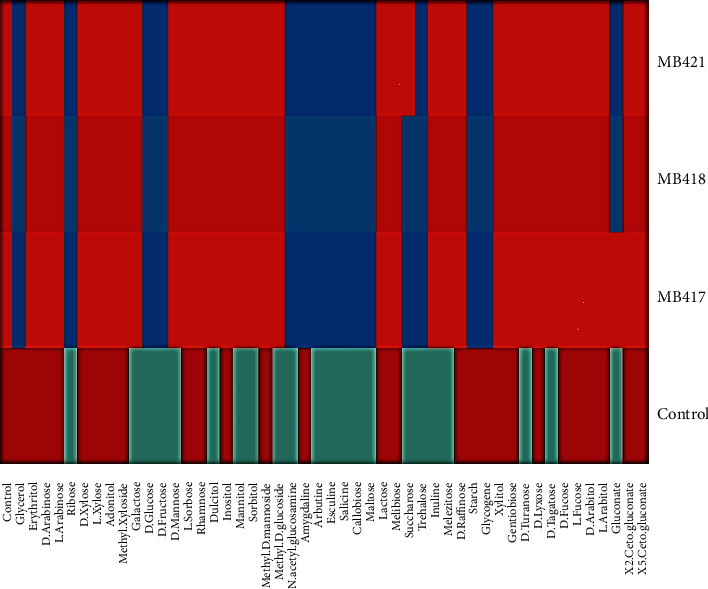
API 50 CH fermentation patterns of fermented olive isolate MB417, MB418, and MB421 along with *Lactobacillus bulgaricus* as a reference strain, where blue color fields represent positive test results and red field represents negative test results.

**Figure 3:**
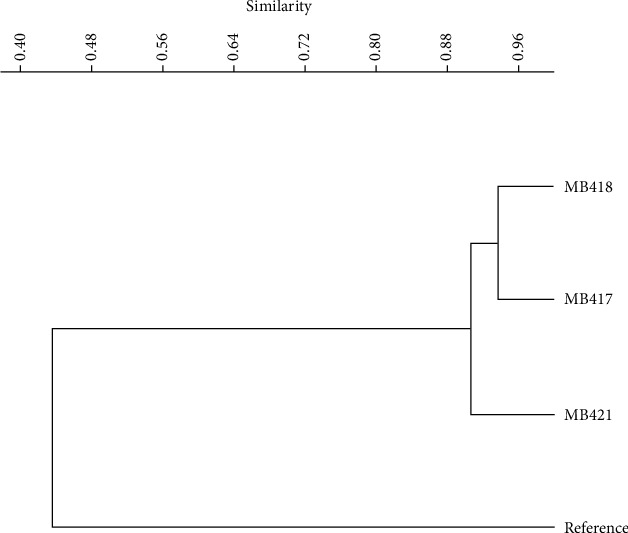
Similarity index of probiotic bacteria from fermented black and green olives.

For *Lactobacillus crispatus, Lactococcus lactis,* and *Carnobacterium divergens*, the results of biochemical tests showed characteristics similar to those of reported probiotics, for example, not being catalase producers (anaerobes or facultative anaerobes that only ferment and do not respire using oxygen as a terminal electron acceptor), being nonspore formers, and having a thick peptidoglycan cell wall structure (gram-positive). The isolates ferment glucose during anaerobic incubation as well as during aerobic incubation, but no gas was produced during the 24 h of incubation, both under aerobic and anaerobic conditions (Table 2). Whereas the isolates were identified by carbohydrate fermentation patterns on the API 50 CH panel, the carbohydrate fermentation ability of the isolated strains was analyzed against frothy nine different sugars (Figure 2). The heat map summarizes the sugar fermentation pattern of probiotic bacteria from fermented olives and groups the strains based on similarities and differences in the sugar fermentation profile shown in Figure 3. The species-level identification was done using API web50 CHL v5.1 (Table 3).

### 3.2. Characterization of Optimal Temperature and pH

**Figure 4:**
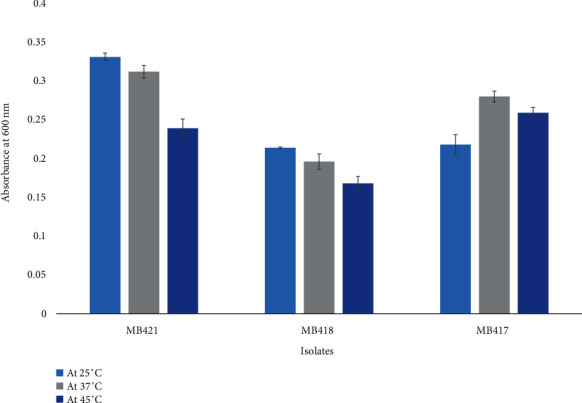
Growth of isolated bacteria from fermented black and green olives at different temperatures.

**Figure 5:**
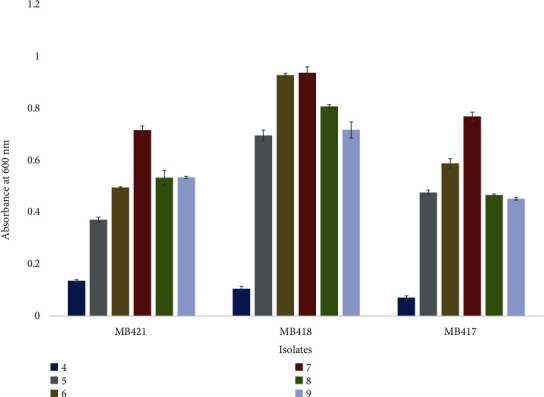
Growth of bacterial isolates from black and green fermented olives at various pH.

*Carnobacterium divergens* MB421 from green olives and *Lactoccocus lactis* MB418 from black olives grew optimally at 25°C, and *Lactobacillus crispatus* MB417 showed optimal growth at 37°C (Figure 4). Optimal growth of *Carnobacterium divergens* MB421, *Lactobacillus crispatus* MB417, and *Lactoccocus lactis* MB418 was observed at pH 7, showing the neutrophilic behavior of the isolates (Figure 5).

### 3.3. Evaluation of Antibiotic Susceptibility Profile

The antibiotic susceptibility of isolates to various antibiotic discs by disc diffusion assay was determined in terms of standard inhibitory zones, and the results are shown in Table 4. All the isolated strains were sensitive to imipenem. *Lactobacillus crispatus* MB417 and *Carnobacterium divergens* MB421 showed resistance to amoxicillin.

### 3.4. Antimicrobial Activity

The isolates were assessed for antimicrobial properties and tested negative on nutrient agar medium against *Bacillus cereus* MB401, *Streptomyces laurentii* MB319, *Klebsiella pneumoniae* MB081, and *Acaligens facelis* MB090.

On Mueller-Hinton (MH) agar medium, isolates behaved differently as compared to nutrient agar medium. This might be due to the MH medium, which is the standard medium for the performance of such tests in microbiology laboratories. *Lactobacillus crispatis* MB417 and *Lactococcus lactis* MB418 isolated from black olives were sensitive to *Streptomyces laurentii* MB319. Isolates depicted no inhibitory zone against *Microbacterium oxydans* MB325*, Klebsiella pneumoniae* MB081, and *Acaligens facelis* MB090, but showed antimicrobial activity against *Bacillus cereus* MB401, *Bacillus subtilis* MB405 and *Streptomyces laurentii* MB319. The growth of *Enterococcus faecium* JH22 and *Enterococcus facelis* OGRE1 was tested on Brain Heart Infusion (BHI) agar medium. *Carnobacterium divergens* MB421 produced an inhibitory zone against *Enterococcus facelis* OGRE1.

### 3.5. Bile Salt Tolerance Assay

**Figure 6:**
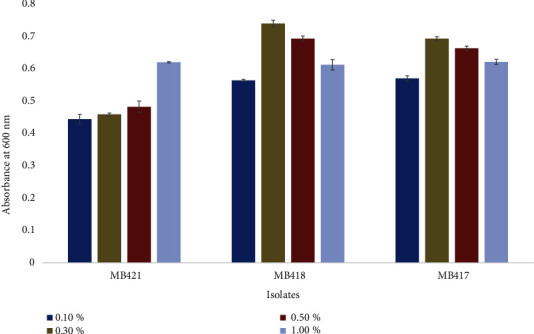
Growth of isolated probiotic bacteria from black and green olives at different bile salt concentrations.

Isolated probiotics were able to tolerate 0.1-1% bile salt and flourish at 0.3% gastrointestinal bile salt concentration (Figure 6**)**. The isolates *Lactobacillus crispatus* MB417 and *Lactococcus lactis* MB418 grew optimally at 0.3% bile salt concentration with a gradual decrease to higher concentrations that were 0.5% and 1.0%, whereas *Carnobacterium divergens* MB421 showed a linearly increasing growth trend starting from 0.1 to 1% bile salt concentration.

### 3.6. Tolerance to Simulated Gastric Juice

**Figure 7:**
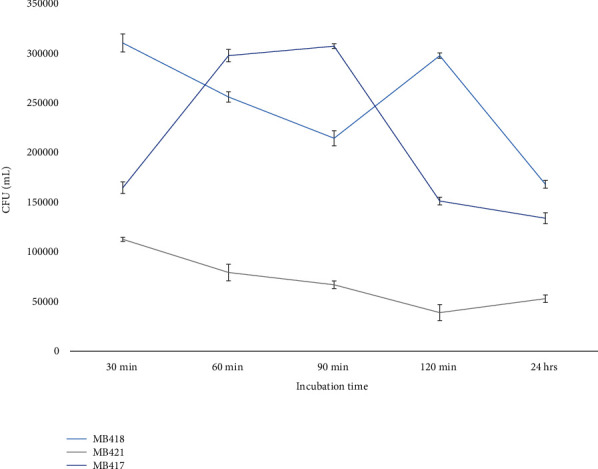
CFU/ml of fermented green and black olives isolates for gastric juice tolerance test.

Potential probiotic bacterial isolates were able to tolerate the acidic pH (2) of stimulated gastric juice through the course of incubation, but some isolates were unable to withstand such harsh conditions in the gut. Colony-forming units per milliliter (CFU/ml) were calculated for black and green olive isolates (Figure 7). *Carnobacterium divergens* MB421 showed a gradual reducing trend during incubation inside the artificially stimulated gastric juice; while *Lactococcus lactis* MB18 showed the same reducing viability trend till 90 min of incubation (might be the acclimatization time). The viability count increased at 120 min of incubation and decreased when measured after 24 h of incubation. *Lactobacillus crispatus* MB417 showed a parabolic endurance pattern peaking at 90 min of incubation in simulated gastric juice.

### 3.7. Salt Tolerance

Following the assessment of salt tolerance, isolates were shown to be resistant to high NaCl concentrations (Table 5), even at a 9% concentration of salt, especially the black olive isolate *Lactobacillus crispatus* MB417. Isolates *Lactococcus lactis* MB418 and *Carnobacterium divergens* MB421 endure up to 8% and 7% NaCl concentrations, respectively.

### 3.8. Quantification of Organic Acid Production

All three isolates exhibited the capability to coagulate skim milk and produce organic acid under gradually decreasing pH (Table 6). Against 0.1 M NaOH, *Lactobacillus crispatus* MB417 produced elevated organic acids during 24 to 72 h. Similar results were shown by *Carnobacterium divergens* MB421, but in the case of *Lactococcus lactis* MB418 decreased with time, measured at 24, 48, and 72 h of incubation. The organic acid production ability of residing potential probiotic bacteria inside the brine (in which fermented olives were stored) was assayed during research work, that is, at the beginning (1st stage), in the middle (2nd stage), and at the end of practical work (3rd stage). During incubation, the pH and acidity of the brine remained unchanged; this could be because of the refrigerated storage of the fermented olives (Table 7).

## 4. Discussion

The present study was conducted to isolate and characterize the potential probiotic isolates from fermented black and green olives. Table olives are the best source of probiotic bacteria [53]. Table olives are considered functional foods because of their nutritional value related to the presence of phenolic compounds and monounsaturated fatty acids [54].

The oxidation-fermentation test in this study identified that the isolated bacteria as facultative anaerobes about their ability to perform fermentation in aerobic as well as anaerobic conditions. The results indicated that all the isolates were able to conduct fermentation during aerobic conditions. All the isolates ferment lactose to produce acid, which in turn changes the color of the medium from green to yellow, showing the isolates were fermenters. Positive fermentation ability was also reported by other scientists on lactic acid bacteria from table olives [55–57] and lactic acid bacterial isolates from yogurt [14].

The study showed that most of the isolates were mesophiles, as they prefer 37 °C, but some of the isolates were slightly thermophilic. Isolates from another study on green Algerian olives demonstrated tolerance to temperatures ranging from 15 to 45°C [57, 58].

In the case of the isolation of probiotics from yogurt, isolates could tolerate pH up to 2.5 with good growth, but according to the current study, after pH 4, growth was very low. This was most likely because isolates from yogurt or dairy origins are more adapted to low pH than isolates from vegetables and meat (due to the source's lactose content, which is then converted to lactic acid after fermentation) [14, 59]. *Lactobacillus crispatus* MB417, *Lactococcus lactis* MB418, and *Carnobacterium divergens* MB421 showed good growth at pH 7, depicting their mesophilic nature. Similarly, mesophilic (pH 6.5-7.5) strains of lactic acid bacteria were reported from various research data [14, 57–59].

The genomes of probiotic bacterial isolates may contain many antimicrobial resistance genes, *van(X)*, *van(E)*, *gyr(A)*, and *tet(M)* genes that code for resistance to the respective vancomycin, ciprofloxacin, and tetracycline antibiotics [60–62]. As a key feature, a good probiotic candidate should not possess or acquire any antibiotic-resistance genes. This research found that most of the isolates were resistant to amoxicillin. This might be because of the wide and nonspecific use of antibiotics that could subsidize the propagation of resistance in the bacterial population used for the fermentation of olives [63]. Along with this, in Pakistan, the use of unprescribed antibiotics is a common practice, which results in the development of resistance to many of the pathogenic bacteria from antibiotics, including amoxicillin. Whereas, it was also reported that all of the probiotic isolates were sensitive to streptomycin, imipenem, and chloramphenicol.

Out of a total of 60 combinations of tests for bacteriocin production activity of six isolates against 10 different indicator bacteria, only two gave positive results on nutrient agar medium. This finding was comparable with some other reported probiotic strains, *L. caseishirota*, *L. paracasei* sub spp. *tolerans*, *L. plantarum* [64, 65], and *L. fermentum* [66, 67], which also did not produce bacteriocin against other bacteria. These results concluded that there is no existence of bacteriocin-like action [68], and inhibition of surrounding microbes was due to the acidic environment produced by probiotic LAB strains [69].

The present study showed that all isolates were able to tolerate salt concentrations up to 7%, but few were able to tolerate high concentrations. All isolates showed maximum growth with a 1% NaCl concentration, which is consistent with the findings of other studies [14, 70].

In the present study, 0.1, 0.3, 0.5, and 1% bile salt concentrations were used for the growth of potential probiotic isolates. In a healthy human, 0.3% bile salt is present in the GIT, and for any bacteria to be used in probiotic production, it must tolerate this bile concentration [14, 52, 71, 72]. All of the black olive isolates and a few of the green olive isolates gave maximum growth on 0.3% bile salt, and almost all of the isolates tolerate this bile concentration (up to 1%). This was because of a vital characteristic of *Lactobacilli* which enabled them to survive, grow, and exert their action in the GIT due to the action of bile salt hydrolase, thus reducing the toxic side effects of bile salts. Along with this, some components of food protect and promote resistance among strains to bile salts [68].

To achieve health benefits, probiotic foods must comprise an adequate amount of live bacteria, at least 10^6-7^ CFU/g [73]. The ability to tolerate harsh conditions and viable cell counts of isolated potential probiotic bacteria were checked while incubating in artificial gastric juice with pH 2.2 for 30, 60, 90, 120 minutes, and 24 h. The experiments yielded ≤10^5^ bacterial counts, and recent studies have provided significant data on the valuable immunological effects derived from dead probiotic cells [68]. The viability of isolates from black olives (*Lactoccocus lactis* MB418) and green olives (MB422) gradually decreased with incubation time, while black olive isolates MB416, and *Lactobacillus crispatus* MB417; and green olive isolates MB424, MB422 showed stability up to 24 h of incubation in acidic gastric juice. Results of the experiment predict that gastric juice in the GIT has fewer effects that could be hostile to most of the probiotic isolates [74]. Similar viability counts (3.4-5.6 logs CFU/ml) for the fermented olive isolates from the present study were also observed previously for *L. plantarum, L. pentosus,* and *L. paraplantarum*, showing 3-6 log CFU/ml viable bacterial cells [68].

In natural olives, organic acids can be added to create an optimal initial pH for the proliferation of LAB [69, 75]. Olive samples used in the study also contained added lactic, ascorbic, and citric acids before packaging. The organic acid production ability of probiotic bacteria present inside the brine of black and green olives was determined to have an idea of the shelf life of the fermented food, which can be spoiled in terms of taste, texture, and smell due to altered lactic acid production. The Acidity of the brine of black and green olives remained the same throughout, but the brine of green olives was more acidic with a lower pH as compared to black olives. Because green olives were un-ripened, the bacteria employed for their fermentation had to work more to make them less bitter than for fully ripened black olives, for example, maturation of olives will condition phenolic compounds, sugar, and cell wall permeability [69].

In our study, the data analysis showed that one of the isolates, MB422, from green olives, is the least similar to the rest of the isolates. Isolates from black olives MB416 and green olives *Carnobacterium divergens* MB421 showed 84% similarity, which is 72% similar to *Lactococcus lactis* MB418. The cluster is 60% similar to other clusters, such as MB424 and *Lactobacillus crispatus* MB417 (75% similar).

## 5. Conclusion

It is concluded that isolated potential probiotic bacteria are well adapted to the olive surface or brine. Thus, the olives, either green or black, contained good microbial flora of probiotics and could be consumed as an effective probiotic food. As a result, the isolates from our study, specifically MB417, MB418, MB421, and MB424, demonstrated relatively good antipathogenic activity and survival in harsh conditions, suggesting that they could be considered viable “next-generation” probiotic candidates, which could be beneficial to the pharmaceutical industry.

### 5.1. Future Applications

It would be advantageous to conduct a detailed study on the identification of isolates using molecular methods. The use of bacteriocin producers as starters is of considerable interest because bacteriocin production is a significant factor that helps to promote the safety and quality of fermented table olives and can also be used as natural antibiotics.

## Figures and Tables

**Table 1:** List of isolated bacteria from fermented green and black olives on MRS medium.

Source	Isolates	
	Black olives	Green olives
Chunks	MB414	MB419
		MB420
Suspension	MB415	MB421
	MB416	MB422
	MB417	MB423
Brine	MB418	MB424
		MB425

Suspension: suspension from minced olives.

**Table 2:** Identification of probiotic bacteria isolated from black and green olives.

Strains	Morphology		Biochemical tests						Aerobic condition		Anaerobic condition	
	Staining	Shape	S F	Cat	M R	V P	I P	S C	Growth	A P	Growth	A P
*Lactobacillus crispatus* MB417	+	Bacilli	—	*—*	+w	—	—	*—*	*++*	*+*	*++*	*+*
*Lactococcus lactis* MB418	+	Cocci	—	*—*	—	+w	—	*—*	*++*	+	++	+
*Carnobacterium divergens* MB421	+	Bacilli	—	*—*	+w	+w	—	—	*++*	*+*	*++*	*+*

+Gram positive: S F: spore formatio; Cat: catalase production; M R: methyl red; V P: Voges-Proskauer; I P: indole production; S C: Simmon's citrate; A P: acid production.

**Table 3:** Carbohydrates fermentation pattern of probiotic isolates from fermented black and green olives on API 50 CH panel.

API 50 tests	*Lactobacillus crispatus* MB417	*Lactococcus lactis* MB418	*Carnobacteriumdivergens* MB421
Salicine	+	+	+
Potassium gluconate	+	+	+
N-Acetyl glucosamine	+	+	+
L-Sorbose	—	—	—
L-arabinose	—	—	—
Inuline	—	—	—
Glycerol	V	+	+
Gentiobiose	V	V	—
Esculine/iron citrate	+	+	+
D-Turanose	—	—	—
D-Trehalose	+	+	+
D-tagatose	—	—	—
D-Saccharose	+	—	+
D-ribose	+	+	+
D-Rafinose	—	—	—
D-Melezitose	—	—	—
D-Melbiose	—	—	—
D-Mannitol	—	—	—
D-Maltose	+	+	+
D-Lactose	—	—	—
D-Glucose	+	+	+
D-Galactose	—	—	—
D-Fructose	+	+	+
D-Cellobiose	+	+	+
Arbutine	+	+	+
Amygdaline	+	+	+

**Table 4:** Antibiotic resistance/sensitivity profile of isolated probiotic bacteria from black and green against different antibiotics.

Name of antibiotics	Resistance	Intermediate	Sensitive	Profiling pattern of antibiotics for the three isolates		
				Sensitivity %	Intermediate %	Resistance %
AMC	≤13	14-16	≥17	0	0	100
CN	≤12	13-14	≥15	100	0	0
S	≤11	12-14.5	≥15	100	0	0
TE	≤14	15-18	≥19	66.6	33.3	0
K	≤13	14-17	≥18	100	0	0
IMP	≤15	16-20	≥21	100	0	0
C	≤12	13-17	≥18	100	0	0
B	≤10	11-13.5	≥14	0	66.6	33.3
E	≤13	14-22	≥23	33.3	66.6	0
N	≤12	13-15	≥16	66.6	33.3	0

AMC: amoxicillin: CN: gentamicin; S: streptomycin; TE: tetracycline; K: kanamycin; IMP: imipenem; C: chloramphenicol; B: bacitracin; E: erythromycin; N: neomycin; ^∗^All zones were measured in millimeters.

**Table 5:** Tolerance of different concentrations of NaCl by isolated probiotic bacteria isolated from black and green olives.

Strains	Salt concentration					
	1%	3%	5%	7%	8%	9%
*Lactobacillus crispatus* MB417	++	++	+	+	+	+w
*Lactococcus lactis* MB418	++	++	+	+	+	—
*Carnobacterium divergens* MB421	++	++	+w	+w	—	—

++ = maximum growth; + = normal growth; +w = weak growth, - = negative growth.

**Table 6:** Results for quantification of organic acid production by isolated probiotic bacteria from black and green olives.

Strains	Milk coagulation	Incubation time (24 hours)		Incubation time (48 hours)		Incubation time (72 hours)	
		pH	Organic acid (%)	pH	Organic acid (%)	pH	Organic acid (%)
*Lactobacillus crispatus* MB417	+	6.09	1.126	4.54	7.208	4.35	10.812
*Lactococcus lactis* MB418	+	4.96	5.406	5.52	3.604	5.45	2.703
*Carnobacterium divergens* MB421	+	6.07	1.802	4.53	5.406	4.26	9.01

+ = positive test result.

**Table 7:** Determination of organic acid production in a brine of black and green olives through the course of time during the study period.

Brine	At beginning		During middle		At end	
	pH	Organic acid (%)	pH	Organic acid (%)	pH	Organic acid (%)
Green olives	3.56	6.307	3.6	5.406	3.62	7.208
Black olives	5.67	0.901	5.72	0.901	5.76	0.901

## Data Availability

All the data is available in the manuscript.

## References

[1] Bandyopadhyay B., Mandal N. C. (2014). Probiotics, prebiotics and synbiotics-in health improvement by modulating gut microbiota: the concept revisited. *International Journal of Current Microbiology and Applied Sciences*.

[2] Nya E. J., Uffia I. D. (2021). Bacterial inhibition and antioxidant activity of probiotic yoghurt developed using microbial isolates from soymilk. *European Journal of Biology and Biotechnology*.

[3] Malik S. S., Saeed A., Baig M., Asif N., Masood N., Yasmin A. (2018). Anticarcinogenecity of microbiota and probiotics in breast cancer. *International Journal of Food Properties*.

[4] Scholz-Ahrens K. E., Adolphi B., Rochat F. (2016). Effects of probiotics, prebiotics, and synbiotics on mineral metabolism in ovariectomized rats -- impact of bacterial mass, intestinal absorptive area and reduction of bone turn-over. *Nfs Journal*.

[5] Ranadheera R. D., Baines S. K., Adams M. C. (2010). Importance of food in probiotic efficacy. *Food Research International*.

[6] Oleskin A. V., Shenderov B. A. (2019). Probiotics and psychobiotics: the role of microbial neurochemicals. *Probiotics And Antimicrobial Proteins*.

[7] Clarke G., Cryan J. F., Dinan T. G., Quigley E. M. (2012). Review article: probiotics for the treatment of irritable bowel syndrome – focus on lactic acid bacteria. *Alimentary Pharmacology & Therapeutics*.

[8] Taye Y., Degu T., Fesseha H., Mathewos M. (2021). Isolation and identification of lactic acid bacteria from cow milk and milk products. *The Scientific World Journal*.

[9] Fariq A., Saeed A. (2016). Production and biomedical applications of probiotic biosurfactants. *Current Microbiology*.

[10] Kelesidis T., Pothoulakis C. (2012). Efficacy and safety of the probiotic saccharomyces boulardii for the prevention and therapy of gastrointestinal disorders. *Therapeutic Advances in Gastroenterology*.

[11] Morrow L. E., Gogineni V., Malesker M. A. (2012). Probiotics in the intensive care unit. *Nutrition in Clinical Practice*.

[12] Pais P., Almeida V., Yılmaz M., Teixeira M. C. (2020). Saccharomyces boulardii: what makes it tick as successful probiotic?. *Journal of Fungi*.

[13] Ni H., Raikos V. (2019). Lactic-acid bacteria fermentation-induced effects on microstructure and interfacial properties of oil-in-water emulsions stabilized by goat-milk proteins. *Lwt*.

[14] Hoque M. Z., Akter F., Hossain K. M., Rahman M. S. M., Billah M. M., Islam K. M. D. (2010). Isolation, identification and analysis of probiotic properties of lactobacillus spp. from selective regional yoghurts. *World journal dairy food*. *Science*.

[15] Tavares L. M., De Jesus L. C., Da Silva T. F. (2020). Novel strategies for efficient production and delivery of live biotherapeutics and biotechnological uses of Lactococcus lactis: the lactic acid bacterium model. *Frontiers in Bioengineering and Biotechnology*.

[16] Elbanna K., El Hadad S., Assaeedi A., Aldahlawi A., Khider M., Alhebshi A. (2018). In vitro and in vivo evidences for innate immune stimulators lactic acid bacterial starters isolated from fermented camel dairy products. *Scientific Reports*.

[17] Park Y. H., Hamidon F., Rajangan C. (2016). Application of probiotics for the production of safe and high-quality poultry meat. *Korean Journal for Food Science of Animal Resources*.

[18] Alves M., Peres C. M., Hernandez-Mendonza A., Bronze M. R., Peres C., Malcata F. X. (2015). Olive paste as vehicle for delivery of potential probiotic *Lactobacillus plantarum* 33. *Food Research International*.

[19] Ziadi M., Bouzaiene T., Lakhal S. (2019). Screening of lactic starter from Tunisian fermented vegetables and application for the improvement of caper (Capparis spinosa) fermentation through an experimental factorial design. *Annals of Microbiology*.

[20] Aymerich T., Rodríguez M., Garriga M., Bover-Cid S. (2019). Assessment of the bioprotective potential of lactic acid bacteria against *Listeria monocytogenes* on vacuum-packed cold-smoked salmon stored at 8 °C. *Food Microbiology*.

[21] Anacarso I., Messi P., Condò C. (2014). A bacteriocin-like substance produced from *Lactobacillus pentosus* 39 is a natural antagonist for the control of *Aeromonas hydrophila* and *Listeria monocytogenes* in fresh salmon fillets. *LWT-Food Science and Technology*.

[22] Mathur H., Field D., Rea M. C., Cotter P. D., Hill C., Ross R. P. (2017). Bacteriocin-antimicrobial synergy: a medical and food perspective. *Frontiers in Microbiology*.

[23] Tapiba V., Nasr N., Higazy A. M. (2015). Isolation, identification and application of bacteriocin-like inhibitory substances producing bacterial strains. *Interntionl Journl Current Microbiolgy Applied Sciences*.

[24] Besnard G., Cheptou P. O., Debbaoui M. (2020). Paternity tests support a diallelic self-incompatibility system in a wild olive (Olea europaea subsp. laperrinei, Oleaceae). *Ecology and Evolution*.

[25] Sebastiani L., Busconi M. (2017). Recent developments in olive (Olea europaea L.) genetics and genomics: applications in taxonomy, varietal identification, traceability and breeding. *Plant Cell Reports*.

[26] Corsetti A., Perpetuini G., Schirone M., Tofalo R., Suzzi G. (2012). Application of starter cultures to table olive fermentation: an overview on the experimental studies. *Frontiers in Microbiology*.

[27] Reboredo-Rodríguez P., González-Barreiro C., Martínez-Carballo E. (2021). Applicability of an in-vitro digestion model to assess the bioaccessibility of phenolic compounds from olive-related products. *Molecules*.

[28] Fernández-Poyatos M. P., Ruiz-Medina A., Llorent-Martínez E. J. (2019). Phytochemical profile, mineral content, and antioxidant activity of *Olea europaea* L. cv. Cornezuelo table olives. Influence of *in vitro* simulated gastrointestinal digestion. *Food Chemistry*.

[29] Nikfarjam A. K., Mahdian E., Ghavidel R. A., Karazhyan R. (2021). Probiotic potential of lactobacillus strains isolated from Iranian traditional pickled garlic and using them in fermented olive. *Journal of BioScience and Biotechnology*.

[30] Lavermicocca P., Angiolillo L., Lonigro S. L. (2018). Lactobacillus plantarum 5BG survives during refrigerated storage bio-preserving packaged Spanish-style table olives (cv. Bella di Cerignola). *Frontiers in Microbiology*.

[31] Aprile A., Negro C., Sabella E. (2019). Antioxidant activity and anthocyanin contents in olives (cv cellina di nardò) during ripening and after fermentation. *Antioxidants*.

[32] Heperkan D. (2013). Microbiota of table olive fermentations and criteria of selection for their use as starters. *Frontiers in Microbiology*.

[33] Doulgeraki A. I., Pramateftaki P., Argyri A. A., Nychas G. J. E., Tassou C. C., Panagou E. Z. (2013). Molecular characterization of lactic acid bacteria isolated from industrially fermented Greek table olives. *LWT-Food Science and Technology*.

[34] Begum R., Sarker M. A. K., Islam M. A., Alam M. K., Pramanik M. K. (2017). Isolation and characterization of lactic acid bacteria from indigenous dairy product and preparation of starter culture by freeze-drying. *Bioresearch Communications-(BRC)*.

[35] Mahato S., Shahani A. K. (2019). Identifying the Diversity of Dominant LABs from Fermented Dairy Products Dahi and Yoghurt in Eastern Region of Nepal. *Journal of Food Science and Technology Nepal*.

[36] Çelekli A., Alslibi Z. A., Bozkurt H. (2020). Boosting effects of Spirulina platensis, whey protein, and probiotics on the growth of microflora and the nutritional value of ayran. *Engineering Reports*.

[37] Gram C. (1884). The differential staining of Schizomycetes in tissue sections and in dried preparations. *Fortschitte der Medicin*.

[38] Mushtaq M., Bukhari S. M., Ahmad S. (2021). Isolation and characterization of bacteria residing in the oral, gut, and fecal samples of different pheasant species. *Brazilian Journal of Biology*.

[39] Harley J. P. (2005). *Laboratory Exercises in Microbiology*.

[40] Jo O., Okobia U. B. (2021). The prevalence of *enterococcus spp.* in toilet door handles of male and female hostel in delta state polytechnic ozoro. *International journal of modern*. *Pharmaceutical Research*.

[41] McDevitt S. (2009). Methyl red and Voges–Proskauer test protocols. *American Society for Microbiology*.

[42] Rosenberg E., Rubinovitz C., Legmann R., Ron E. Z. (1988). Purification and chemical properties of Acinetobacter calcoaceticus A2 biodispersan. *Applied and environmental microbiology*.

[43] Diéguez A. L., Balboa S., Romalde J. L. (2020). *Halomonas borealis* sp. nov. and *Halomonas niordiana* sp. nov., two new species isolated from seawater. *Systematic and Applied Microbiology*.

[44] Lemos M. L., Toranzo A. E., Barja J. L. (1985). Modified medium for the oxidation-fermentation test in the identification of marine bacteria. *Applied and Environmental Microbiology*.

[45] Simatende P., Siwela M., Gadaga T. H. (2019). Identification of lactic acid bacteria and determination of selected biochemical properties in emasi and emahewu. *South African Journal of Science*.

[46] Yang E., Fan L., Yan J. (2018). Influence of culture media, pH and temperature on growth and bacteriocin production of bacteriocinogenic lactic acid bacteria. *AMB Express*.

[47] Wanja D. W., Mbuthia P. G., Waruiru R. M., Bebora L. C., Ngowi H. A., Nyaga P. N. (2020). Antibiotic and disinfectant susceptibility patterns of bacteria isolated from farmed fish in Kirinyaga county, Kenya. *International Journal of Microbiology*.

[48] Contente D., Feito J., Borrero J. (2020). Lactococcus lactis RBT18: from the rainbow trout farm to the lab, the tale of a nisin Z producer. *In Multidisciplinary Digital Publishing Institute Proceedings*.

[49] Nami Y., Vaseghi Bakhshayesh R., Mohammadzadeh Jalaly H., Lotfi H., Eslami S., Hejazi M. A. (2019). Probiotic properties of *enterococcus* isolated from artisanal dairy products. *Frontiers in Microbiology*.

[50] Dunne C., O'Mahony L., Murphy L. (2001). In vitro selection criteria for probiotic bacteria of human origin: correlation with in vivo findings^1,2,3,4^. *The American Journal of Clinical Nutrition*.

[51] Choi S. J., Yang S. Y., Yoon K. S. (2021). Lactic acid bacteria starter in combination with sodium chloride controls pathogenic *Escherichia coli* (EPEC, ETEC, and EHEC) in kimchi. *Food Microbiology*.

[52] Graciela F., Maria P. T., Spencer J. F. T., Spencer A. L. R. D. (2001). *Food Microbiology Protocols*.

[53] Peres C. M., Peres C., Malcata F. X. (2017). Role of natural fermented olives in health and disease. *Fermented Foods in Health and Disease Prevention*.

[54] Buckland G., Gonzalez C. A. (2015). The role of olive oil in disease prevention: a focus on the recent epidemiological evidence from cohort studies and dietary intervention trials. *British Journal of Nutrition*.

[55] Argyri K., Doulgeraki A. I., Manthou E. (2020). Microbial diversity of fermented Greek table olives of Halkidiki and Konservolia varieties from different regions as revealed by metagenomic analysis. *Microorganisms*.

[56] Papadelli M., Zoumpopoulou G., Georgalaki M. (2015). Evaluation of two lactic acid bacteria starter cultures for the fermentation of natural black table olives (Olea europaea L cv. Kalamon). *Polish Journal of Microbiology*.

[57] Yalçınkaya S., Kılıç G. B. (2019). Isolation, identification and determination of technological properties of the halophilic lactic acid bacteria isolated from table olives. *Journal of Food Science and Technology*.

[58] Mourad K., Halima Z. K., Nour-Eddine K. (2004). Isolation of lactic acid bacteria for its possible use in the fermentation of green Algerian olives. *Grasas y Aceites*.

[59] Ashraf R., Smith S. C. (2015). Selective enumeration of dairy based strains of probiotic and lactic acid bacteria. *International Food Research Journal*.

[60] Ashraf R., Smith S. C. (2016). Commercial lactic acid bacteria and probiotic strains-tolerance to bile, pepsin and antibiotics. *International Food Research Journal*.

[61] Lim S. M., Lee N. K., Kim K. T., Paik H. D. (2020). Probiotic *Lactobacillus fermentum* KU200060 isolated from watery kimchi and its application in probiotic yogurt for oral health. *Microbial Pathogenesis*.

[62] Zheng M., Zhang R., Tian X., Zhou X., Pan X., Wong A. (2017). Assessing the risk of probiotic dietary supplements in the context of antibiotic resistance. *Frontiers in Microbiology*.

[63] Manyi-Loh C., Mamphweli S., Meyer E., Okoh A. (2018). Antibiotic use in agriculture and its consequential resistance in environmental sources: potential public health implications. *Molecules*.

[64] Maragkoudakis P. A., Miaris C., Rojez P. (2006). Production of traditional Greek yoghurt using *Lactobacillus* strains with probiotic potential as starter adjuncts. *International Dairy Journal*.

[65] Hosseini N. M., Hussain M. A., Britz M. L. (2015). Stress responses in probiotic Lactobacillus casei. *Critical Reviews in Food Science and Nutrition*.

[66] El-Mohsen S. A., El-Leboudy A. A., Ibrahim H. R., Amer A. A. (2019). Anti-helicobacter pylori activity of lactobacillus fermentum in fermented skimmed bovine Milk. *Alexandria Journal for Veterinary Sciences*.

[67] Lin W. H., Yu B., Jang S. H., Tsen H. Y. (2007). Different probiotic properties for *Lactobacillus fermentum* strains isolated from swine and poultry. *Anaerobe*.

[68] Argyri A. A., Zoumpopoulou G., Karatzas K. A. G. (2013). Selection of potential probiotic lactic acid bacteria from fermented olives by *in vitro* tests. *Food Microbiology*.

[69] Hurtado A., Reguant C., Bordons A., Rozès N. (2012). Lactic acid bacteria from fermented table olives. *Food Microbiology*.

[70] Soni M., Shah H. R., Patel S. M. (2021). Isolation, identification and analysis of probiotic characteristics of lactobacillus spp. from regional yoghurts from Surendranagar District, Gujarat. *Asian Journal of Dairy and Food Research*.

[71] Liu W., Chen M., Duo L. (2020). Characterization of potentially probiotic lactic acid bacteria and bifidobacteria isolated from human colostrum. *Journal of Dairy Science*.

[72] Munoz-Quezada S., Chenoll E., Vieites J. M. (2013). Isolation, identification and characterisation of three novel probiotic strains (lactobacillus paracaseiCNCM I-4034,Bifidobacterium breveCNCM I-4035 andLactobacillus rhamnosusCNCM I-4036) from the faeces of exclusively breast-fed infants. *British Journal of Nutrition*.

[73] Cui L., Niu L.-y., Da-jing L. I. (2018). Effects of different drying methods on quality, bacterial viability and storage stability of probiotic enriched apple snacks. *Journal of Integrative Agriculture*.

[74] Piatek J., Gibas-Dorna M., Olejnik A. (2012). The viability and intestinal epithelial cell adhesion of probiotic strain combination-in vitro study. *Annals of Agricultural and Environmental Medicine*.

[75] Perpetuini G., Prete R., Garcia-Gonzalez N., Khairul Alam M., Corsetti A. (2020). Table olives more than a fermented food. *Food*.

